# Preclinical* In Vitro* and* In Vivo* Evaluation of [^18^F]FE@SUPPY for Cancer PET Imaging: Limitations of a Xenograft Model for Colorectal Cancer

**DOI:** 10.1155/2018/1269830

**Published:** 2018-02-13

**Authors:** T. Balber, J. Singer, N. Berroterán-Infante, M. Dumanic, L. Fetty, J. Fazekas-Singer, C. Vraka, L. Nics, M. Bergmann, K. Pallitsch, H. Spreitzer, W. Wadsak, M. Hacker, E. Jensen-Jarolim, H. Viernstein, M. Mitterhauser

**Affiliations:** ^1^Biomedical Imaging and Image-Guided Therapy, Division of Nuclear Medicine, Medical University of Vienna, Vienna, Austria; ^2^Department of Pharmaceutical Technology and Biopharmaceutics, Faculty of Life Sciences, University of Vienna, Vienna, Austria; ^3^Institute of Pathophysiology and Allergy Research, Center of Pathophysiology, Infectiology and Immunology, Medical University of Vienna, Vienna, Austria; ^4^Department of Internal Medicine II, University Hospital Krems, Karl Landsteiner University of Health Sciences, Krems an der Donau, Austria; ^5^Department of Radiation Oncology, Division of Medical Physics, Medical University of Vienna, Vienna, Austria; ^6^Comparative Medicine, The Interuniversity Messerli Research Institute, The University of Veterinary Medicine Vienna, Medical University of Vienna, and University of Vienna, Vienna, Austria; ^7^Department of Nutritional Sciences, Faculty of Life Sciences, University of Vienna, Vienna, Austria; ^8^Department of Surgery, Surgical Research Laboratories, Medical University of Vienna, Vienna, Austria; ^9^Institute of Organic Chemistry, University of Vienna, Vienna, Austria; ^10^Department of Pharmaceutical Chemistry, Faculty of Life Sciences, University of Vienna, Vienna, Austria; ^11^CBmed GmbH, Graz, Austria; ^12^Ludwig Boltzmann Institute Applied Diagnostics, Vienna, Austria

## Abstract

Molecular imaging probes such as PET-tracers have the potential to improve the accuracy of tumor characterization by directly visualizing the biochemical situation. Thus, molecular changes can be detected early before morphological manifestation. The A_3_ adenosine receptor (A_3_AR) is described to be highly expressed in colon cancer cell lines and human colorectal cancer (CRC), suggesting this receptor as a tumor marker. The aim of this preclinical study was the evaluation of [^18^F]FE@SUPPY as a PET-tracer for CRC using* in vitro* imaging and* in vivo* PET imaging. First, affinity and selectivity of FE@SUPPY and its metabolites were determined, proving the favorable binding profile of FE@SUPPY. The human adenocarcinoma cell line HT-29 was characterized regarding its hA_3_AR expression and was subsequently chosen as tumor graft. Promising results regarding the potential of [^18^F]FE@SUPPY as a PET-tracer for CRC imaging were obtained by autoradiography as ≥2.3-fold higher accumulation of [^18^F]FE@SUPPY was found in CRC tissue compared to adjacent healthy colon tissue from the same patient. Nevertheless, first* in vivo* studies using HT-29 xenografts showed insufficient tumor uptake due to (1) poor conservation of target expression in xenografts and (2) unfavorable pharmacokinetics of [^18^F]FE@SUPPY in mice. We therefore conclude that HT-29 xenografts are not adequate to visualize hA_3_ARs using [^18^F]FE@SUPPY.

## 1. Introduction

Colorectal cancer (CRC) is the fourth leading cause of cancer-related deaths in men and women worldwide [1]. The primary diagnosis is usually made by colonoscopy and biopsy, which often does not reflect the full extent of the disease due to tumor heterogeneity and disregard of potential metastases. Positron Emission Tomography (PET) provides a noninvasive imaging technique, which is valuable for tumor staging and clinical decision making and to estimate the patient's prognosis [2]. Besides the routinely used PET-tracer [^18^F]FDG, the availability of specific tumor tracers would enhance the characterization of colorectal tumors and help in CRC staging and with the choice of treatment.

An essential characteristic of most solid tumors is hypoxia, which inevitably leads to accumulation of adenosine within the tumor microenvironment as a result of the breakdown of adenine nucleotides, which has been recognized in the 1990s [3, 4]. Since then, many efforts have been made to clarify the role of adenosine and its receptors in cancer [5–7]. The expression of the A_3_ adenosine receptor (A_3_AR), which is one of four subtypes of the adenosine receptor family, has been reported in several human tumor cell lines including leukemia (Jurkat T, HL-60), melanoma (A375), and astrocytoma (ADF) [8–12]. In particular, there is a rising interest in the involvement of A_3_ARs in CRC as A_3_AR protein expression has been reported for various colon cancer cell lines, including Caco-2, HCT-116, CCL-228, DLD-1, and HT-29 [13–15]. Merighi et al. have shown that caffeine leads to hypoxia-inducible factor-1 (HIF-1) protein accumulation and increased vascular endothelial growth factor (VEGF) expression through A_3_AR stimulation in HT-29 cells under hypoxic conditions [16]. According to Sakowicz-Burkiewicz et al., treatment with the A_3_AR agonist IB-MECA (1 *μ*M) results in an A_3_AR-dependent growth promoting effect in HT-29 cells. In contrast, IB-MECA causes cell apoptosis in HCT-116 cells, similarly in an A_3_AR dependent manner [13].

High expression of A_3_AR mRNA and protein has been reported in colon and breast carcinoma compared to adjacent nonneoplastic tissue by Madi et al. Remarkably, even higher levels of A_3_AR mRNA have been found in lymph node metastases than in primary tumor tissue, suggesting A_3_AR-overexpression as a marker for tumor progression [17]. Additionally, Gessi et al. studied A_3_AR expression in colorectal cancer tissue samples of 73 patients and provided evidence that the A_3_AR has the potential to be used as a diagnostic marker for colon cancer. The authors have shown ≥2-fold increased A_3_AR protein expression in primary colon carcinomas compared to normal mucosa and describe a tendency towards higher A_3_AR expression in large adenomas compared to small adenomas. Therefore, the authors proposed a major role of the A_3_AR in cancer aggressiveness [18]. Moreover, radioligand binding experiments using the A_3_AR antagonist [^3^H]MRE 3008F20 and western blot analysis indicated that the A_3_AR is the most abundant of all four adenosine receptor subtypes in colorectal cancer tissues as well as in colon cancer cell lines (Caco-2, DLD-1 and HT-29). On the contrary, RT-PCR experiments showed relatively low levels of A_3_AR mRNA in the mentioned colon cancer cell lines compared to mRNA levels of the other adenosine receptor subtypes [15]. As mRNA levels do not necessarily correlate with protein levels [19] and protein transcription is a prerequisite for targeted receptor imaging approaches such as PET imaging, protein expression data is the most relevant for this study.

The A_3_AR antagonist [^18^F]FE@SUPPY has been presented as the first PET-tracer for hA_3_AR imaging in 2008 by Wadsak et al. [20, 21]. First preclinical PET imaging using CHO-K1-hA_3_AR xenografts has shown promising results leading to further evaluation of this PET-tracer in oncology [22]. Besides [^18^F]FE@SUPPY and [^18^F]FE@SUPPY:2, only a few other PET-ligands have been proposed for A_3_AR imaging, including carbon-11 labeled 1,2,4-triazolo[4,3-a]quinoxalin-1-one derivatives and bromine-76 labeled nucleoside ligands ([^76^Br]MRS3581 and [^76^Br]MRS5147) [23–25]. To our knowledge, no preclinical* in vivo* PET imaging has been reported for these A_3_AR PET-ligands so far. In our preclinical study, we aimed to evaluate [^18^F]FE@SUPPY as a PET-tracer for human cancer using* in vitro* imaging and* in vivo* PET imaging in a CRC tumor model.

## 2. Methods

### 2.1. General

#### 2.1.1. Cell Culture

HT-29 cells and CHO-K1 cells were purchased from ATCC. HT-29 cells were cultured in RPMI 1640 medium supplemented with 10% fetal calf serum, 2 mM L-glutamine, and 10 *μ*g/mL gentamicin sulfate. Human A_3_ adenosine receptor-expressing CHO-K1 cells (CHO-K1-hA_3_AR) were purchased from PerkinElmer (ValiScreen® GPCR cell line) and were grown using Ham's F12 supplemented with 10% FCS, 2 mM L-glutamine, penicillin (100 U/M), streptomycin (100 *μ*g/mL), and 0.4 mg/mL G418. Parental CHO-K1 cells were cultured likewise, but without selection antibiotics. Cells were maintained under standard conditions in a humidified incubator (37°C, 5% CO_2_).

#### 2.1.2. Animals

Six-week-old male BALB/c mice (BALB/cAnNRj, Division of Laboratory Animal Science and Genetics, Himberg, Austria) were kept under conventional housing conditions, with food and water supply ad libitum and a 12 h day/night cycle. Male, immunodeficient CB17-SCID mice (CB-17/Icr-*Prkdc*^scid^/Rj, Janvier Labs, France) of the same age were kept under specific pathogen-free conditions in individually ventilated cages. All animals were treated according to the European Union rules on animal care. The corresponding animal experiments were approved by the Austrian Ministry of Sciences (BMWFW-66.009/0031-WF/V/3b/2015, BMWFW-66.009/0029-WF/V/3b/2015).

#### 2.1.3. Tumor Grafting

After 10 to 14 days upon arrival, CB17-SCID mice were injected subcutaneously with 2 × 10^6^ HT-29 cells into one flank and 2 × 10^6^ CHO-K1 cells in the opposite flank (*n* = 9). Body weight and tumor development were monitored every second day by caliper measurement. The respective tumor volume was calculated according to the following equation: tumor volume (mm^3^) = *d*^2^ ×* D/2* (where* d* is the shortest diameter and* D* the longest diameter). Animals were subjected to *μ*PET imaging 10 days after inoculation, when tumors reached a volume of at least 300 mm^3^. Tumor volume never exceeded 1 cm^3^.

#### 2.1.4. Human Tissues

Colorectal carcinoma tissue and adjacent healthy colon tissue were obtained directly after tumorectomy from two patients after full informed consent and quick-frozen in 2-methylbutane (−40°C). Tissue was sliced into 16 *μ*m slices using a microcryotome (Thermo Scientific Microm HM 560) and stored at −80°C until usage. Depending on the sample size, 3 to 4 different regions were defined and analyzed by means of autoradiography and immunohistochemistry.

### 2.2. Characterization of Binding and Target Expression

#### 2.2.1. Competitive Binding Assay

Competitive binding assays were performed using hA_1_AR, hA_2A_AR, or hA_3_AR expressing cell membranes (18.5 ng/*μ*L, 16.7 ng/*μ*L, or 1.7 ng/*μ*L final protein concentration, resp.) and 1.7 nM [^3^H]DPCPX (*K*_*D*_ = 1.7 nM), 50 nM [^3^H]CGS21680 (*K*_*D*_ = 23 nM), or 0.4 nM [^125^I]AB-MECA (*K*_*D*_ = 0.78 nM) as the respective radioligands (all purchased from PerkinElmer, Inc. Waltham, USA). The assay was performed according to the manufacturer's instructions in a final volume of 500 *μ*L. Increasing concentrations of test compounds were added, whereby the concentration of dimethyl sulfoxide (DMSO) in final assay volume remained ≤10% (hA_1_AR and hA_2A_AR assay) and ≤1% in the hA_3_AR assay. Nonspecific binding was determined using 1 *μ*M DPCPX (hA_1_R assay), 1 *μ*M SCH-442,416 (hA_2A_R assay), or 10 *μ*M I-AB-MECA (hA_3_AR assay). Filtration through GF/B filters (Whatman®, presoaked in 0.1% PEI or 0.5% BSA) was performed using a cell harvester (Brandel®), and receptor-bound radioactivity was determined via gamma counting (2480 Wizard^2^, PerkinElmer) or liquid scintillation counting (Hidex 300 SL). IC_50_ fitted binding curves were generated using the GraphPad Software 5.0, and *K*_*i*_ values were calculated using the Cheng-Prusoff equation.

#### 2.2.2. Flow Cytometry

For the flow cytometric evaluation of hA_3_AR expression, single-cell suspensions of HT-29 cells (2 × 10^5^ per tube) were fixed and permeabilized using Cytofix/Cytoperm™ kit (BD Biosciences). Cells were incubated with mouse monoclonal anti-human A_3_AR (100 *μ*L of 4 *μ*g/mL in PBS + 2% FCS, Abnova H00000140-M01) or mouse IgG2b kappa isotype control (100 *μ*L of 4 *μ*g/mL in PBS + 2% FCS, eBioscience™ 14-4732-85) for 1 h at 4°C. Following a washing step, bound primary antibodies were detected with rabbit anti-mouse IgG FITC (100 *μ*L of 40 *μ*g/mL in PBS + 2% FCS, Dako F0261) for 30 min at 4°C in the dark. Samples were analyzed on a FACSCalibur™ flow cytometer (BD Bioscience), whereby 10,000 single cells were recorded.

#### 2.2.3. Western Blot

Cell lysates were prepared from 75 cm^2^ cell culture flasks when cells reached 80% confluency using radioimmunoprecipitation assay (RIPA) buffer and protease inhibitor cocktail according to the manufacturer's instructions. Tissue lysates from HT-29 xenografts were prepared according to a standard protocol using RIPA buffer (according to sample size approx. 4 times of lysis buffer), protease inhibitor, and Ultra-Turrax® for homogenization. The protein concentration of cell lysates was determined using Pierce™ BCA Protein Assay Kit (Thermo Scientific), and 20 *μ*g protein per well was loaded onto TGX™ precast gels (Bio-Rad). After gel electrophoresis (200 V, 30 min), proteins were transferred to nitrocellulose membranes (Amersham™ Protran™ Premium 0.2 *μ*m NC, GE Healthcare Life Sciences) via semidry blotting (80 mA per gel). Membranes were incubated with rabbit polyclonal anti-A_3_AR (Santa Cruz Biotechnology, Inc. sc-13938) (1 : 750, 2 h, RT) and further incubated with goat anti-rabbit IgG HRP conjugate (1 : 5000, 1 h, RT). Detection was performed using the dedicated kit (SuperSignal West Pico Chemiluminescent Substrate detection kit, Thermo Scientific), and chemiluminescence imaging was conducted (Bio-Rad VersaDoc™ Imaging System).

### 2.3. *In Vitro* Imaging

#### 2.3.1. Immunofluorescence Microscopy

HT-29 cells were seeded on chamber slides (3 × 10^5^/mL, 200 *μ*L per well, 8 well slides) and incubated at 37°C until 50% confluency was reached. Cells were then fixed (4% paraformaldehyde in PBS, 15 min, 4°C), permeabilized (0.2% Triton X in PBS, 2 min, room temperature (RT)), and blocked (2% FCS in PBS, 30 min, RT). Mouse monoclonal anti-human A_3_AR (Abnova H00000140-M01) and mouse IgG2b kappa isotype control (eBioscience 14-4732-85) were used 1 : 50 in PBS + 2% FCS and incubated for 1 h at RT. Cells were washed three times with PBS and incubated with the secondary antibody (rabbit anti-mouse IgG FITC, Dako F0261) for 1 h at RT. After washing, cells were incubated with DAPI (1 : 5000) for 10 min at RT, and subsequently, slides were embedded with an aqueous mounting medium (Fluoromount™, Sigma F4680). Slides were recorded on an Axioplan II fluorescence microscope (Carl Zeiss Microscopy).

#### 2.3.2. Autoradiography

Tissue slices were thawed and reconstituted in assay buffer (50 mM Tris-HCl pH 7.4, 100 mM NaCl, 1 mM EDTA, 1% BSA, 1 unit adenosine deaminase/100 mL) for 30 min at RT. Radiosynthesis of [^18^F]FE@SUPPY was performed as previously described and the product was physiologically formulated (EtOH/0.9% saline 10/90) [20]. Tissue slices were incubated with 50 kBq [^18^F]FE@SUPPY (40–200 GBq/*μ*mol) in 100 *μ*L assay buffer for 1 h at RT. Slices were thoroughly washed with ice-cold wash buffer (50 mM Tris-HCl pH 7.4), dried, and exposed to a phosphor screen overnight. The readout of the phosphor storage screen was performed on a Cyclone Phosphor Imager (Perkin Elmer), and data analysis was performed using OptiQuant® Software as previously described [26]. Statistical testing was performed using GraphPad Prism 5.0 Software. Differences among groups (colorectal cancer versus healthy colon) were analyzed using a two-tailed, unpaired Student's *t*-test with Welch's correction.

#### 2.3.3. Immunohistochemistry

Vicinal cryosections of colorectal carcinoma and healthy colon tissue were stained to identify regions with hA_3_AR expression following a standard protocol. In brief, cryosections were fixed (96% ethanol, 10 min), permeabilized (0.2% Triton X-100 in PBS, 5 min), and blocked using Bloxall™ Blocking Solution and a dedicated avidin/biotin blocking kit (Invitrogen, Thermo Fisher Scientific). Additionally, sections were incubated with goat serum (1 : 10 in PBS) to reduce nonspecific binding. Rabbit polyclonal anti-A_3_AR (1 : 100, ab203298; Abcam) was used 1 : 100 in PBS + 0.1% BSA for 1 h in a humid, dark chamber. Purified rabbit IgG (Life technologies) was used as an isotype control likewise. Cryosections were washed 3 times for 5 min (PBS + 0.1% Tween-20) and incubated with biotinylated anti-rabbit IgG (1 : 200, PBS + 5% goat serum) for 30 min. After washing, further detection was performed with the Vectastain® ABC kit (Vector Laboratories) according to the manufacturer's instructions. DAB substrate kit (Abcam) was used as a chromogen to detect peroxidase, and haematoxylin was used for counterstaining of cell nuclei. Immunohistochemically stained slides were acquired on an automated TissueFAXS microscope system (TissueGnostics, Vienna, Austria) at a 5-fold and 20-fold magnification.

### 2.4. Tracer Stability in Mice

#### 2.4.1. *In Vitro* Stability Tests

Stability of [^18^F]FE@SUPPY was tested against mouse liver microsomes, mouse S9 fraction, and mouse plasma (BD Sciences). Amount of intact tracer (%) was determined after 5, 10, 20, 40, 60, and 120 min using an Agilent series 1100/1200 HPLC system connected to a radioactivity detector (Raytest, Ramona Star) (*n* = 2 in triplicate). The assay was conducted as previously described for respective rat and human enzymes [27].

#### 2.4.2. *Ex Vivo* Blood Stability

Radiosynthesis was performed as described elsewhere [20] and the product was processed as follows: ethanol was totally evaporated and the dry product was again physiologically formulated in 1.5–2 mL Tween-20/EtOH/0.9% saline 1/9/90 to obtain activity concentrations of approximately 1 GBq/mL. Healthy BALB/c mice were injected with 18 ± 2 MBq (molar activity = 70–200 GBq/*μ*mol) retroorbitally and sacrificed after 5, 10, 20, 40, and 70 min (*n* = 3 for each time point). Blood samples were collected and immediately precipitated using acetonitrile/methanol (10 : 1) and centrifuged (12,000 rpm, 5 min, 4°C). The obtained supernatants were subjected to radio-HPLC as previously described [22].

### 2.5. Biodistribution

Ex vivo biodistribution of [^18^F]FE@SUPPY was assessed 70 min after tracer application in BALB/c mice. Radioactivity was determined using a gamma counter (2480 Wizard^2^, PerkinElmer), organs were wet-weighted, and percentage of injected dose per gram of organ was calculated (%ID/g).

### 2.6. *In Vivo* Imaging

Xenograft-bearing CB17-SCID mice were anesthetized using isoflurane (2.5%) mixed with oxygen (1.5 L/min) to avoid movement during the imaging. Blocking agents (2 mg/kg BW FE@SUPPY or MRS1523) or the respective vehicle control (Tween-20/EtOH/0.9% physiological saline 1/9/90) was administered retroorbitally 2 min prior to the radiotracer administration (*n* = 3 per group). Subsequently, the animals received another retroorbital injection of 17.42 ± 4.5 MBq [^18^F]FE@SUPPY into the venous plexus of the opposite eye. With a minor delay after the application of the radiotracer (2-3 min), mice were placed into the field of view of the scanner (*μ*PET/CT Inveon, Siemens Medical Solution, Knoxville, USA), and dynamic imaging was performed for 60 min to follow tracer distribution. Vital parameters (respiration, body temperature) were continuously monitored using a dedicated monitoring unit (bioVet; m2m imaging, Cleveland, OH, USA) to ensure the depth of anesthesia and wellbeing of the animals. Retroorbital application volumes did not exceed 100 *μ*L per application.

## 3. Results and Discussion

### 3.1. Characterization of Binding and Target Expression

Affinity and selectivity of FE@SUPPY and its potential metabolites upon cleavage by carboxylesterases, DFE@SUPPY, and FE@SUPPY:11 [28] were determined in competitive binding assays. FE@SUPPY has been first described by Li et al., who reported a *K*_*i*_ value of 4.22 ± 0.7 nM for human A_3_AR. However, this study only provided the selectivity ratio towards rat A_1_AR (rA_1_AR/hA_3_AR = 7400) [29]. Here, we confirmed the affinity of FE@SUPPY towards the human A_3_AR (*K*_*i*_ = 6.02 ± 0.4 nM, *n* = 3) and demonstrated its selective hA_3_AR binding compared to the other human adenosine receptors. Moreover, the respective theoretical metabolites show little affinity for the hA_3_AR, supporting the potential of FE@SUPPY as a ligand for human* in vivo* application (Table 1).

**Figure 1:**
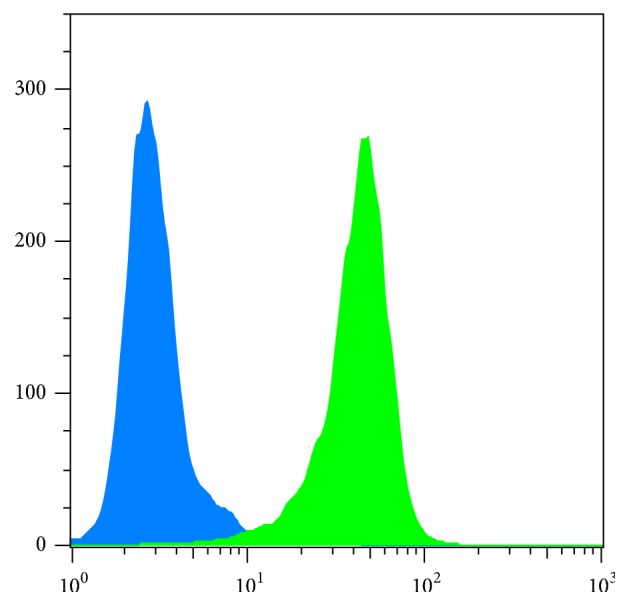
Flow cytometric analysis of HT-29 cells revealed expression of hA_3_AR protein (green). The isotype control did not show fluorescence signal (blue).

The human colorectal adenocarcinoma cell line (HT-29) was characterized regarding its hA_3_AR protein expression using flow cytometry and western blot. Flow cytometric analysis resulted in mean fluorescence intensity (ΔMFI) of 53.6 ± 22 in three independent experiments (Figure 1). Additionally, A_3_AR protein expression in HT-29 cells was determined by western blot (Figure 8) (western blot results are discussed separately below). This is in line with previous studies, which reported A_3_AR expression for this cell line as well [13, 15]. Thus, HT-29 cells were subsequently chosen for tumor graft experiments.

### 3.2. *In Vitro* Imaging

**Figure 2:**
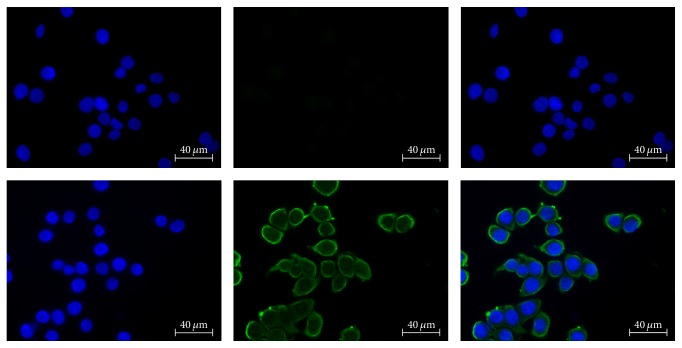
Immunofluorescent staining of HT-29 cells; left: DAPI, middle: FITC, right: merge. Upper row: mouse isotype control, lower row: anti-human A_3_AR staining.

Fluorescence microscopy of HT-29 cells showed cell membrane-specific staining, pointing at the expression of hA_3_AR on the cell surface, which is typical for GPCRs (Figure 2).

**Figure 3:**
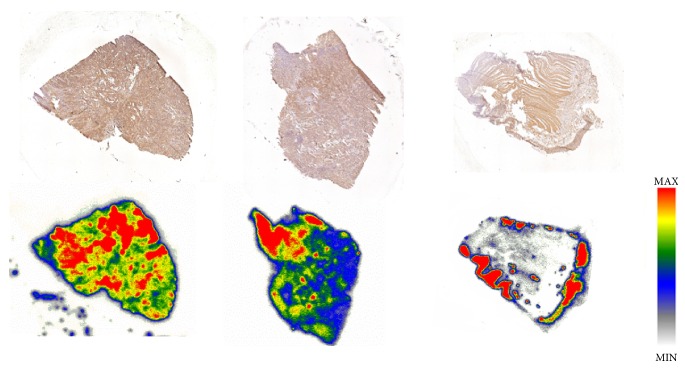
Upper row: immunohistochemical staining of hA_3_AR. Lower row: autoradiographic analysis of the corresponding vicinal tissue slices using [^18^F]FE@SUPPY. Left and center: colon cancer tissue, right: healthy colon tissue.

In all investigated regions of the two CRC patients, [^18^F]FE@SUPPY accumulation was higher in colorectal carcinoma tissue slices than in healthy colon tissue slices of the same individual (for detailed analysis see supplementary (available here)). In 5 of 7 regions, a ≥2.3-fold higher binding of [^18^F]FE@SUPPY was found (*P* < 0.05). This finding is in accordance with Gessi et al., who reported similar ratios by means of [^3^H]MRE 3008F20 binding [18]. Regions with high accumulation of [^18^F]FE@SUPPY corresponded to regions with high hA_3_AR expression identified by immunohistochemistry (Figure 3).

### 3.3. Tracer Stability in Mice

**Figure 4:**
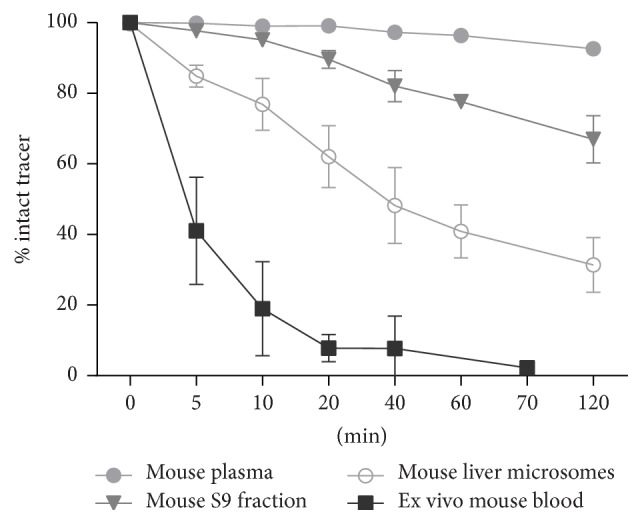
*In vitro *stability and* ex vivo *blood stability of [^18^F]FE@SUPPY.

**Figure 5:**
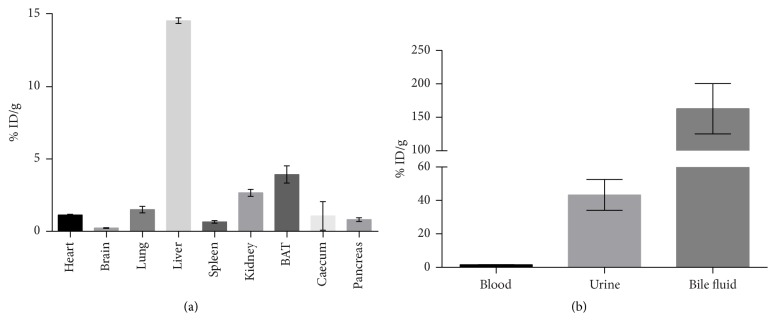
Biodistribution of [^18^F]FE@SUPPY in healthy BALB/c mice (*n* = 3). (a) shows biodistribution in organs. (b) shows accumulation in body liquids.

[^18^F]FE@SUPPY exhibited high stability in mouse plasma, as 92.6 ± 0.7% of intact tracer could still be detected after 120 min of incubation at 37°C.* In vitro* stability tests against mouse liver homogenates (S9 fraction) and purified mouse liver microsomes showed 66.9 ± 6.7% and 31.4 ± 7.8% intact tracer after 120 min, respectively. Ex vivo blood stability analysis demonstrated more rapid degradation of [^18^F]FE@SUPPY* in vivo* than that observed in* in vitro* testing as only 2.2 ± 0.4% intact [^18^F]FE@SUPPY could be determined after 70 min (Figure 4). This data indicates higher metabolism in mice compared to rats described in previously conducted studies, where 25.8 ± 5.3% intact tracer was found in plasma after 60 min [22]. However, these data could also be mimicked by the fact that intact [^18^F]FE@SUPPY is rapidly cleared from blood hepatobiliary (into the bile fluid, compare Figures 5 and 7), and the equilibrium in blood is therefore shifted to the metabolites.

### 3.4. Biodistribution

Biodistribution was assessed 70 min after tracer application in healthy BALB/c mice and revealed a high accumulation of radioactivity in fat-rich regions (brown adipose tissue, BAT) likely due to the tracer's lipophilicity [30]. Regarding the emunctory organs, liver showed the highest accumulation (14.57 ± 0.20% ID/g), followed by the kidneys (2.67 ± 0.24% ID/g). The additional analysis of body liquids pointed at a mainly hepatobiliary excretion of [^18^F]FE@SUPPY, as the highest amount was found in bile fluid (162.78 ± 37.51% ID/g). The amount of radioactivity in the kidneys and urine (43.33 ± 9.23% ID/g) suggests the excretion of the hydrophilic radioactive metabolite, 2-[^18^F]fluoroethanol [31], which was already proposed by Haeusler et al. [28]. The circulating radioactivity in blood was low after 70 min (1.6 ± 0.1% ID/g). This finding is in accordance with the results obtained by the* ex vivo* blood analysis. Moreover, pronounced accumulation of [^18^F]FE@SUPPY was found in A_3_AR rich tissues such as the heart (1.13 ± 0.04% ID/g) and lung (1.50 ± 0.23% ID/g). A similar biodistribution pattern was observed for rats in a previously conducted study [20]. [^18^F]FE@SUPPY accumulation in the brain was low after 70 min (0.23 ± 0.03% ID/g) (Figure 5).

### 3.5. *In Vivo* Imaging

**Figure 6:**
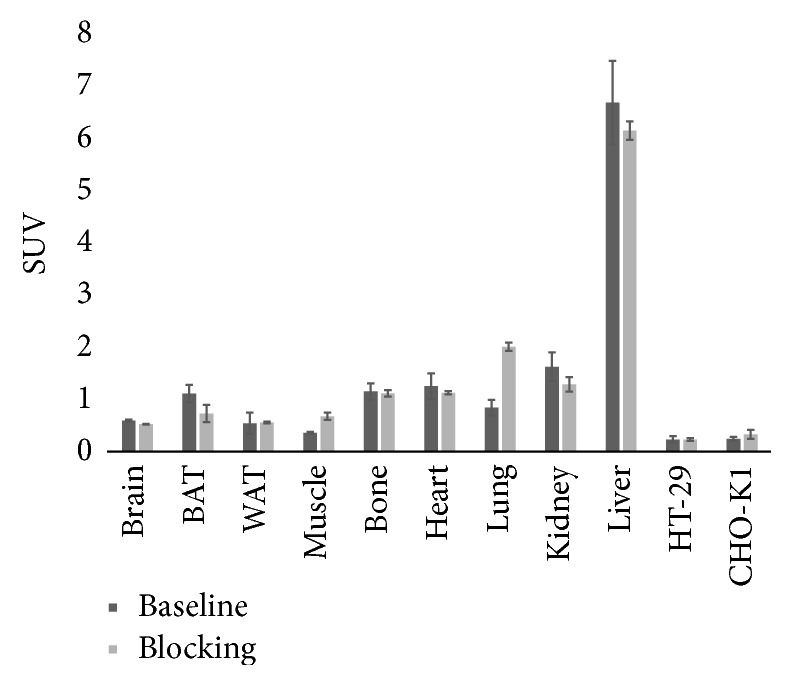
*μ*PET-imaging was performed for 60 min. Activity concentration of [^18^F]FE@SUPPY in organs of interest is expressed as standardized uptake value (SUV). Blocking experiments, shown in the figure, were performed using unlabeled FE@SUPPY. Blocking experiments using MRS1523 provided the same outcome and are not shown in the figure.

*μ*PET imaging of the mouse xenograft model revealed high uptake of [^18^F]FE@SUPPY in the emunctory organs, which was again most pronounced in the liver (SUV = 6.68 ± 0.80). Low standardized uptake values were observed in tumor masses of both HT-29 and CHO-K1 xenograft tumors (SUV = 0.23 ± 0.06 and 0.25 ± 0.33), respectively. There was no difference between CHO-K1 xenografts, which served as a negative control (human A_3_AR negative), and HT-29 xenografts. Moreover, significant blocking could not be achieved (Figure 6). The affinity of FE@SUPPY for the mouse A_3_AR is uncertain but is expected to be lower than that for the human A_3_AR due to the known species differences. The lack of adequate rodent models, mainly due to the low affinity of most hA_3_AR ligands to the rodent A_3_AR, was already recognized by Yamano et al. who proposed a humanized mouse model [32]. Specific uptake was therefore not expected in mouse tissues. Interestingly, a significant influence of the blocking was observed in BAT (decrease in uptake) and lung (increase in uptake). However, the data is based on a set of three individuals in each group, and displacement was not performed in the same individuals. Since tumor uptake in the chosen model was insufficient and not blockable, this phenomenon was not investigated any further.

**Figure 7:**
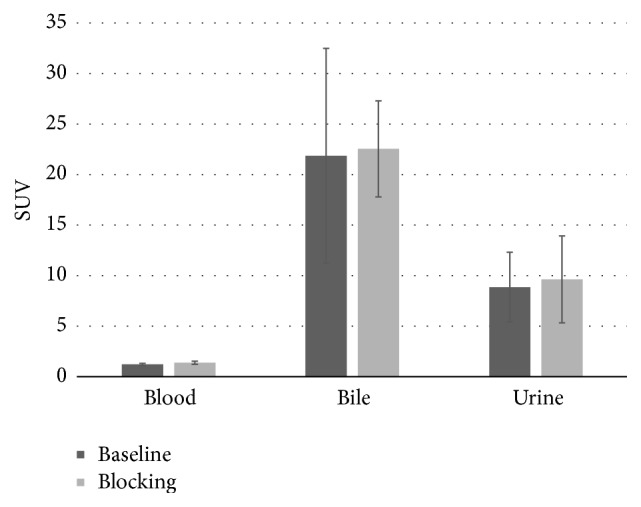
*μ*PET-imaging was performed for 60 min. Activity concentration of [^18^F]FE@SUPPY in body liquids is expressed as standardized uptake value (SUV). Blocking experiments, shown in the figure, were performed using unlabeled FE@SUPPY. Blocking experiments using MRS1523 provided the same outcome and are not shown in the figure.

For a detailed analysis of the pharmacokinetics, volumes of interest were also generated for mouse body liquids including blood, urine, and bile fluid (Figure 7). The radioactivity in blood was generally low (SUV = 1.21 ± 0.11) compared to the body liquids, urine (SUV = 8.86 ± 3.44), and bile fluid (21.85 ± 10.63), showing the highest accumulation of [^18^F]FE@SUPPY, which is in line with the biodistribution experiments. The mentioned standardized uptake values refer to baseline conditions.

**Figure 8:**
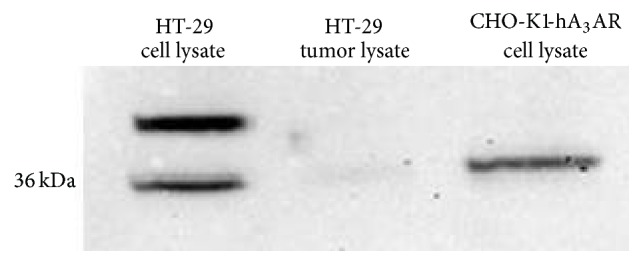
Western blot analysis of HT-29 cell lysate and tissue lysate derived from HT-29 xenograft tumors. CHO-K1-hA_3_AR cell lysate was loaded as a positive control. An additional bond of unknown identity was detected in HT-29 cell lysate.

Adenosine concentrations of ~0,5 *μ*M have been proposed in HT-29 tumors grown as xenografts [4]. Even though adenosine displays only intermediate affinity for the A_3_AR (~1 *μ*M at the rat A_3_AR [33]), the PET-tracer would have to compete with the endogenous ligand for A_3_AR occupancy. This may decrease accumulation of [^18^F]FE@SUPPY in the xenografts. However, more importantly, despite the fact that western blot analysis demonstrated hA_3_AR expression in HT-29 cells, hA_3_AR protein could not be detected in tissue lysates derived from HT-29 xenografts. This indicates that the human receptor is poorly conserved in mice upon tumor graft (Figure 8). To our knowledge, this phenomenon has not been described in literature so far but has tremendous impact on* in vivo* imaging. PET imaging is only feasible if an abundant amount of the target is available, as only nanomolar or even lower concentrations of PET-tracers are applied.

## 4. Conclusion

We found a favorable binding profile of [^18^F]FE@SUPPY displaying high affinity for the human A_3_AR besides low affinity for the other human adenosine receptor subtypes. Autoradiography showed ≥2.3-fold higher uptake in human CRC compared to adjacent healthy colon tissues. First* in vivo* studies using HT-29 xenografts showed insufficient tumor uptake. After initial high expression rates of the A_3_AR in the HT-29 cells, tumor masses, derived from HT-29 xenografts, revealed low target expression. The receptor was not conserved in the xenograft, which hampered the PET imaging strategy. An additional drawback of the used mouse model is the unfavorable pharmacokinetics of the PET-tracer [^18^F]FE@SUPPY in mice. It is questionable how accurate xenograft models in immunocompromised mice are to study the role of human A_3_ARs in cancer. Despite all efforts,* in vivo* visualization of the A_3_AR has not been successful to date and deeper understanding of A_3_AR function is still missing.

## Figures and Tables

**Table 1:** Affinity and selectivity data of FE@SUPPY and metabolites towards adenosine receptor subtypes (*n* = 3–5 in triplicate; amount of DMSO never exceeded 1% of total assay volume in hA_3_AR assay; DMSO was added up to 10% in hA_1_AR and hA_2A_AR assay;^*∗*^*n* = 2 in triplicate; exact *K*_*i*_ value could not be determined due to limited solubility).

Compound	hA_1_AR	hA_2A_AR	hA_3_AR	hA_1_/hA_3_AR	hA_2A_/hA_3_AR
FE@SUPPY	4.03 ± 1.0 *µ*M	1.72 ± 0.4 *µ*M	6.02 ± 0.4 nM	669	285
DFE@SUPPY	5.46 ± 0.4 *µ*M	37.13 ± 16 *µ*M	2.58 ± 1.2 *µ*M	324	112
FE@SUPPY:11	≥57 *µ*M^*∗*^	5.86 ± 0.8 *µ*M	2.80 ± 1.4 *µ*M	≥20	2

## Supplementary Materials

Supplementary Materials Table S1: binding values of FE@SUPPY are expressed as fmol/mm^2^ tissue.
