# Supplementary material for: Preclinical In Vitro and In Vivo Evaluation of [18F]FE@SUPPY for Cancer PET Imaging: Limitations of a Xenograft Model for Colorectal Cancer
Source: Contrast Media Mol Imaging. 2018 Feb 13;2018:1269830. doi: 10.1155/2018/1269830 (PMC5830979; doi:10.1155/2018/1269830)
Supplement: Supplementary Materials — Table S1: binding values of FE@SUPPY are expressed as fmol/mm2 tissue. [file 1269830.f1.pdf]

Supplementary

sample		Colorectal cancer	Healthy colon	P value	ratio
Patient 1	Region 1	7.0 ± 3.6	3.1 ± 0.6	< 0.05	2.3
	Region 2	9.9 ± 1.1	4.1 ± 1.2	< 0.01	2.4
	Region 3	12.2 ± 2.8	4.4 ± 1.2	< 0.01	2.8
	Region 4	7.6 ± 2.3	2.3 ± 0.9	< 0.01	3.3
Patient 2	Region 1	8.6 ± 0.8	3.4 ± 0.2	< 0.01	2.5
	Region 2	8.5 ± 0.9	6.6 ± 1.7	ns	1.3
	Region 3	25.0 ± 2.6	15.6 ± 0.5	< 0.05	1.6

Table S1: Binding values (mean ± SD, n=3-9) of [^{18}F]FE@SUPPY are expressed as fmol/mm^2 tissue.
